# The roles of RRP15 in nucleolar formation, ribosome biogenesis and checkpoint control in human cells

**DOI:** 10.18632/oncotarget.14658

**Published:** 2017-01-14

**Authors:** Zhixiong Dong, Changjun Zhu, Qimin Zhan, Wei Jiang

**Affiliations:** ^1^ Key Laboratory of Molecular and Cellular Systems Biology, Tianjin Normal University, Tianjin 300387, China; ^2^ Tianjin Key Laboratory of Animal and Plant Resistance, College of Life Sciences, Tianjin Normal University, Tianjin 300387, China; ^3^ State Key Laboratory of Molecular Oncology, Cancer Institute and Hospital, Chinese Academy of Medical Sciences and Peking Union Medical College, Beijing 100021, China

**Keywords:** RRP15, nucleolus, ribosome biogenesis, nucleolar stress, checkpoint control

## Abstract

The nucleolus controls ribosome biogenesis and its perturbation induces nucleolar stress that inhibits cell cycle progression and activates checkpoint responses. Here, we investigate the roles of ribosomal RNA processing protein, RRP15, in nucleolar formation, ribosome biogenesis, cell cycle progression and checkpoint control in human cells. RRP15 is localized in the nucleolus and required for nucleolar formation. In contrast to the budding yeast Rrp15p that was reported as a component of pre-60S subunits, RRP15 is found in both pre-40S and pre-60S subunits and involved in regulating rRNA transcription and ribosome biogenesis. Perturbation of RRP15 induces nucleolar stress that activates RPL5/RPL11/5S rRNA (RP)-Mdm2-p53 axis checkpoint response and arrests cells at G1-G1/S in p53-proficient non-transformed RPE1 cells but not in p53-deficient HeLa and MCF7 tumor cells. Instead, p53-deficient HeLa and MCF7 cells with RRP15-dependent nucleolar stress enter S-phase with S-phase perturbation that activates ATR-Chk1- γH2AX axis DNA replication/damage checkpoint response, delaying S-G2/M progression and, ultimately, causing cell death. The selective checkpoint response, cell cycle inhibition and/or cytotoxicity induced by RRP15-dependent nucleolar stress in p53-proficient non-transformed cells and p53-deficient tumor cells suggest that RRP15 might be a potential target for cancer therapy.

## INTRODUCTION

The nucleolus is a sub-organelle that controls ribosome biogenesis in all eukaryotic cells. It is not membrane bound so that the nucleolus can adopt an extremely dynamic structure with a morphology depending on the growth and physiological status of the cell [1, 2]. Light/electronic microscopy, however, reveals that the nucleolus contains three mainly morphologically distinct regions, the fibrillar center (FC), the dense fibrillar component (DFC) and the granular component (GC) [1]. In mammalian cells, several hundred ribosomal DNA genes (rDNA repeats) separated by long intergenic spacers (IGSs) are localized in the FC and are responsible for 47S pre-rRNA transcription [3]. 47S pre-rRNA transcription is dependent on RNA polymerase I (Pol I). After transcription, 47S pre-rRNA transcripts are located to the DFC and subjected to a series of endonucleolytic cleavages and exonucleolytic digestions, termed as pre-rRNA processing. Pre-rRNA processing removes internal and external transcribed spacers (ITSs and ETSs) and generates the mature 18S, 28S, and 5.8S rRNAs. These rRNAs, together with 5S rRNA transcripted by Pol III in the nucleoplasm, bind to small or large subunit ribosomal proteins (RPSs or RPLs) to assemble 40S and 60S pre-ribosomal (pre-40S and pre-60S) subunits in GCs. Subsequently, pre-40S and pre-60S subunits are released into the nucleoplasm and then translocated through nuclear pore complex into the cytoplasm. The final rRNA processing steps occur in the cytoplasm, in which pre-40S and pre-60S subunits mature into 40S and 60S ribosome subunits that assemble into 80S ribosome required for protein translation (for reviews see references [2, 4, 5]).

As ribosome biogenesis is a complex process, it is not surprising that many proteins are involved in this process. Proteomic analyses and individual studies showed that several hundred proteins were localized in the nucleolus and involved in regulating nucleolar formation, structure, function and/or ribosome biogenesis [6–8]. Upstream binding factor (UBF), a Pol I transcriptional activator and enhancer, was found in the FC that bound to entire regions of the rDNA repeats to regulate 47S pre-rRNA transcription. Perturbation of UBF disrupted nucleolar formation and structure, resulting in a significant reduction of 47S pre-rRNA transcription and recruitment of DFC proteins, such as fibrillarin, into the nucleolus [9]. Nucleolin, an abundant nucleolar protein, was involved in regulating rDNA transcription, rRNA maturation and ribosome assembly in the nucleolus [10]. Depletion of nucleolin caused disorganization of nucleolar structure, disrupting transcription elongation of rDNA repeats and dispersing nucleolar proteins from the nucleolus into the nucleoplasm [11–13]. Ribosome proteins (30 RPSs and 49 RPLs) were crucial for assembly of pre-40S and pre-60S subunits [14]. However, despite extensive investigations, the precise functions of many nucleolar proteins, especially ribosomal RNA processing proteins (RRPs), still remain elusive.

Given the fact that ribosome biogenesis controls the protein synthesis in cells, the process is tightly linked with cell proliferation, cell cycle progression and carcinogenesis [1, 15, 16]. Large bodies of evidence indicated that perturbation of nucleolar formation/function and ribosome biogenesis could induce nucleolar stress that inhibited cell cycle progression, activated checkpoint response and promoted carcinogenesis. In mammalian cells, in response to nucleolar disruption/stress, several ribosome proteins including RPL5 and RPL11 were released from the nucleolus into the nucleoplasm where these proteins bound to 5S rRNA to form RPL5/RPL11/5S rRNA complex (RP complex). The RP complex then interacted with p53-directed E3 ubiquitin ligase Mdm2 in the nucleoplasm and inhibited Mdm2 E3 ligase activity to increase p53 protein stability and transcriptional activity in the nucleus, thus activating p53-dependent cell cycle checkpoints [17–20]. Hence, the nucleolar stress-induced RP-Mdm2-p53 axis checkpoint responses blocked cell cycle progression and arrested cells at G1/S and/or G2/M in p53-proficient non-transformed cells for abnormal cell proliferation and transformation protection [12, 21–24]. Consistently, impaired activity/function of the nucleolar stress pathway could be advantageous to cancer cells as it partially alleviated surveillance of ribosome integrity by p53. Thus, like p53, RPL11 and RPL5 were found to be mutated and many ribosomal proteins were overexpressed in human cancers [25, 26]. In addition, several recent studies also showed that perturbation of several ribosomal proteins that induced nucleolar stress in p53-deficient tumor cells could result in tumor cell death, suggesting that nucleolar stress pathway would be an attractive target for cancer therapy although the precise mechanism(s) was unclear [24, 27–29].

In this study, we investigated ribosomal RNA processing protein RRP15 involved in regulating nucleolar formation, ribosome biogenesis, cell proliferation, cell cycle progression and checkpoint control in human non-transformed and cancer cells.

## RESULTS

### RRP15 is a nucleolar protein in human cells

**Figure 1:**
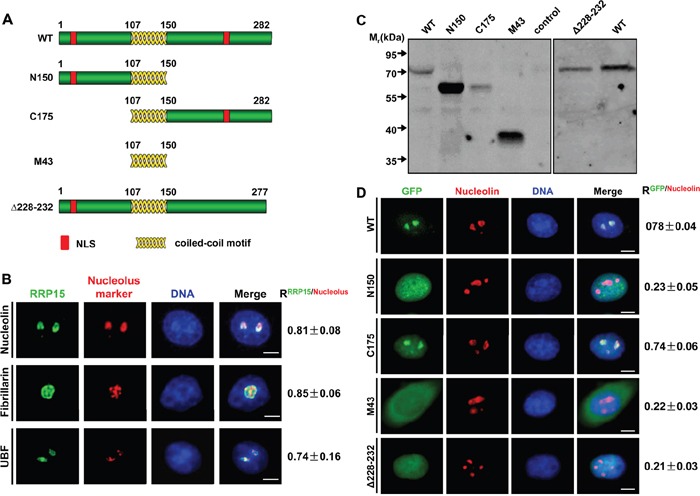
Subcellular localization of RRP15 **A**. The schematic diagrams of RRP15 wild type (WT) and different mutants. **B**. Immunofluorescence analysis of HeLa cells with rabbit α-RRP15 and mouse α-nucleolin, α-fibrillarin or α-UBF. DNA was visualized by DAPI staining. R values were obtained as described (see supplemental Materials and Methods). Scale bars, 5 μm. **C**. HeLa cells were transfected with plasmids of pEGFP-RRP15^WT^, pEGFP-RRP15^N150^, pEGFP-RRP15^C175^, pEGFP-RRP15^M43^ or pEGFP- RRP15^Δ228-232^ for 48 h. Cell lysates were immunoblotted with mouse α-GFP. **D**. HeLa cells grown on coverslips were transfected with plasmids of pEGFP-RRP15^WT^, pEGFP-RRP15^N150^, pEGFP-RRP15^C175^, pEGFP-RRP15^M43^ or pEGFP- RRP15^Δ228-232^ for 48 h. The cells were fixed and stained with mouse α-nucleolin and DAPI. Scale bars, 5 μm. R values were obtained as described in (B).

To characterize human coiled-coil proteins involved in cell cycle control, we employed a large-scale RNA interference (RNAi) screen and identified human ribosomal RNA processing protein 15 (RRP15, also called cgi115). Sequence analysis showed that ribosomal RNA processing protein 15 (Rrp15) was conserved across many species with a coiled-coil motif in the middle of the molecule (Figure 1A and Supplementary Figure 1A). We generated rabbit polyclonal anti-RRP15 antibodies (α-RRP15) to explore the role(s) of RRP15 in human cells. Immunoprecipitation and immunoblotting analyses revealed that α-RRP15, but not pre-bleed sera, specifically recognized 45kDa RRP15 protein (Supplementary Figure 1B).

We examined subcellular localization of endogenous RRP15 using affinity-purified α-RRP15. Immunofluorescence showed that RRP15 was localized predominantly in the nucleolus (Figure 1B), similar to its budding yeast ortholog, Rrp15p, as previously reported [30]. RRP15 was colocalized with nucleolar proteins UBF, fibrillarin, and nucleolin in the nucleoli. To quantitate colocalization of RRP15 with these nucleolar markers, Pearson correlation coefficients (R values) analysis from 30 cells for all pairwise combinations (total positive correlation appears as 1, total negative correlation as -1, and no correlation as 0) revealed that the R value of RRP15/UBF, RRP15/fibrillarin or RRP15/nucleolin was 0.74±0.16, 0.85±0.06 or 0.81±0.08 (Figure 1B). Thus, these results indicated that RRP15 was a nucleolar protein in human cells.

In addition, RRP15 was detected as dispersed nucleoplasmic/cytoplasmic dots when fluorescent imaging intensities were enhanced (Supplementary Figure 2A). We coimmunostained RRP15 with endoplasmic reticulum protein, Bip (a rough ER marker), ribosome proteins RPS6 and RPL11 or α-tubulin (cytoskeletal microtubules) to determine if RRP15 was also a ribosomal component. RRP15 was colocalized with Bip, RPS6 or RPL11 but not with α-tubulin, suggesting that RRP15 might be also involved in ribosome function in the cytoplasm in human cells (Supplementary Figure 2A). Consistent with immunofluorescent results, immunoblotting analysis of subcellular nuclear and cytoplasmic fractions demonstrated that RRP15 was mainly localized in the nucleus and, to a lesser extent, in the cytoplasm (Supplementary Figure 2B).

We detected two potential nuclear localization sequences (NLS) in the N-terminus and C-terminus of RRP15 protein (Figure 1A, red boxes). Analysis of a nucleolar localization sequence (NoLS) detector (http://www.compbio.dundee.ac.uk/www-nod) suggested that the NLS in the C-terminus of RRP15 might be responsible for RRP15 nucleolar localization. To determine if the N-terminal and/or C-terminal NLS of RRP15 were required for RRP15 protein nuclear and/or nucleolar localization, we generated a set of RRP15 mutant proteins fused with green fluorescent protein (GFP) and expressed these proteins (GFP-tagged RRP15 proteins) in HeLa cells using mammalian expression vectors (Figure 1C). Fluorescence imaging and Pearson correlation coefficients of these mutants with nucleolin (R values) were determined. As shown in Figure 1D, deletion of the C-terminus (N150), but not the N-terminus (C175), of RRP15 resulted in perturbation of RRP15 nucleolar localization. Furthermore, deletion of the C-terminal NLS alone (Δ228-232) could sufficiently abrogate RRP15 nucleolar localization (Figure 1D). Taken together, these results indicated that the C-terminal NLS of RRP15 was a functional NoLS required for RRP15 nucleolar localization. In contrast, the N-terminal NLS of RRP15 was only functioned as a NLS, together with the C-terminal NLS of RRP15, required for RRP15 nuclear localization.

### RRP15 is required for nucleolar formation

**Figure 2:**
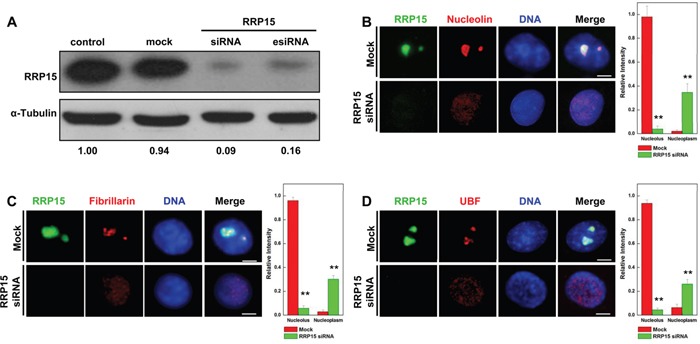
Nucleolar formation and nucleolin, fibrillarin and UBF localization in cells depleted RRP15 **A**. HeLa cells were transfected with mock (buffer), control siRNA, RRP15 siRNA or RRP15 esiRNA for 48 h and cell lysates were immunoblotted with α-RRP15 and anti-α-Tubulin antibody. **B-D**. HeLa cells grown on coverslips were transfected with or without RRP15 siRNA for 48 h, fixed and immunostained with α-RRP15 and α-nucleolin, α-fibrillarin or α-UBF. DNA was visualized by DAPI staining. Scale bars, 5 μm. Intensities of nucleolin, fibrillarin or UBF staining in the nucleoli and the nucleoplasm in 20 cells were determined by Image-Pro Plus 7.0. ** P<0.01.

Subcellular localization of RRP15 suggested that RRP15 could play roles in nucleolar function and/or ribosome biogenesis in human cells. To determine RRP15 functions, we ablated RRP15 in HeLa cells using specific RRP15 small interfering RNAs (siRNAs). Transfection of chemically synthesized RRP15-targeted siRNA (RRP15 siRNA) or endonuclease-prepared RRP15-targeted siRNA (RRP15 esiRNA), but not control siRNA or mock (buffer), significantly reduced endogenous mRNA and protein levels of RRP15 (Figure 2A and Supplementary Figure 3). Immunofluorescence and fluorescence intensities of nucleolar marker nucleolin, fibrillarin or UBF in control cells or cells depleted of RRP15 were determined. As shown in Figure 2B-2D, in contrast to control cells, cells depleted of RRP15 displayed drastic perturbation of nucleolar formation with nucleolin, fibrillarin and UBF dispersing into the nucleoplasm. Consistently, cells depleted of RRP15 also perturbed nucleolar localization of RPL11 and RPS6 that resulted in RPL11 and RPS6 to disperse into the nucleoplasm (Supplementary Figure 4A-4B). However, depletion of RRP15 had little effect on the cytoplasmic localization of RPS6 and RPL11 in these cells (Supplementary Figure 4A-4B). These results indicated that RRP15 was involved in regulating nucleolar formation.

If RRP15 is critical for nucleolar formation, expression of exogenous RRP15, but not its NoLS deletion mutant (Δ228-232), would reestablish the nucleolus in RRP15-depleted cells. Hence, we performed knockdown-rescue experiments, in which HeLa cells were cotransfected with RRP15 esiRNA that targeted the 3′UTR of RRP15 mRNA together with mammalian expression vector expressing GFP-tagged RRP15 or GFP-tagged RRP15^Δ228-232^ using RRP15 cDNA coding region without the 3′UTR. Immunoblotting and immunofluorescence analyses showed that RRP15 esiRNA could effectively ablate the expression of endogenous RRP15 but not the expression of exogenous GFP-tagged RRP15 or GFP-tagged RRP15^Δ228-232^ (Supplementary Figure 5A). Cells expressing GFP-tagged RRP15, but not GFP-tagged RRP15^Δ228-232^, restored nucleolar formation with nucleolin localizing in the nucleolus (Supplementary Figure 5B-5C). Taken together, these results demonstrated that RRP15 was not only a protein localized in the nucleolus but also a protein required for nucleolar formation in human cells.

### RRP15 is constitutes of pre-40S and pre-60S ribosomal subunits and participates in ribosome biogenesis

**Figure 3:**
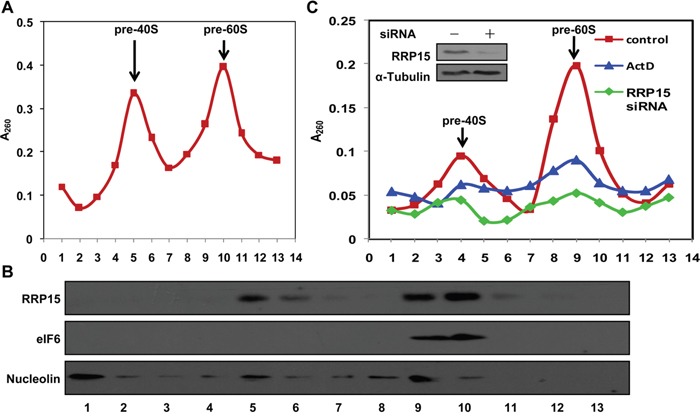
Presentation and regulation of RRP15 in pre-40S and pre-60S ribosomal subunits and ribosome biogenesis **A**. Pre-ribosomal profile in HeLa cells. Nuclear extracts were prepared from HeLa cells and fractionized on 10% to 40% sucrose density gradient. The absorbance at 260 nm (A_260_) of each fraction was profiled and the positions of pre-ribosomal subunits were indicated. **B**. RRP15 in pre-ribosomal subunits. Proteins from fractions described in (A) were separated on a SDS-PAGE and immunoblotted with α-RRP15, α-eIF6 (pre-60S ribosomal subunit protein) and α-Nucleolin (nucleolar protein). **C**. Ribosome biogenesis impaired by RRP15 depletion. HeLa cells transfected with or without RRP15 siRNA for 48 h or HeLa cells were treated with ActD for 12 h (positive control) were collected. Pre-ribosomal subunits were purified from these cells and profiled as described in (A). RRP15 depletion was determined by immunoblotting analysis (insert).

The crucial role of RRP15 in nucleolar formation promoted us to investigate the nucleolar function(s) of RRP15. As one of the primary nucleolar functions in eukaryotic cells was to assemble pre-ribosomal subunits, we examined if RRP15 was involved in assembling pre-40S and/or pre-60S ribosomal subunits in human cells. To this end, we fractionized HeLa cell nuclear extracts to obtain pre-40S and pre-60S ribosomal subunits fractions using a sucrose gradient by ultracentrifugation as previously described [31]. As shown in Figure 3A, analysis of the sucrose gradient fractions by spec-photometer revealed that pre-40S ribosomal subunits presented mainly in the fractions 5 and 6 whereas pre-60S ribosomal subunits presented mainly in the fractions 9 and 10. Immunoblotting analysis of proteins from each sucrose gradient fraction showed that RRP15 presented predominantly in 5, 6, 9 and 10 fractions, indicating that RRP15 was not only a constitute of pre-60S ribosomal subunits as previous studies reported for budding yeast Rrp15p [32], but also a constitute of pre-40S ribosomal subunits in human cells (Figure 3B).

To determine if RRP15 participated in ribosome biogenesis, we ablated expression of RRP15 by RRP15 siRNA and analyzed assembly of pre-40S, pre-60S, 40S, 60S or 80S ribosomal subunits and polysomes in cells depleted of RRP15. As shown in Figure 3C, sucrose gradient analysis indicated that, when compared to control, ablation of RRP15 resulted in significant reductions of pre-40S and pre-60S ribosomal subunits in HeLa cells, similar to the results obtained from HeLa cells treated with actinomycin D (ActD), a RNA polymerase I inhibitor that blocked transcription of 47S pre-rRNA from rDNA repeats and thus affected assembly of pre-40S and pre-60S ribosomal subunits [33]. Furthermore, ablation of RRP15 also resulted in decreased 40S, 60S, 80S and polysomes in cells depleted of RRP15 when compared with control cells (Supplementary Figure 6A). Consistent with these results, immunoblotting analysis revealed that when compared with control cells, protein synthesis profile of GAPDH was significantly decreased in cells depleted of RRP15, similar to cells treated with cycloheximide (CHX) for 8 h (Supplementary Figure 6B and 6C). Taken together, these results indicated that RRP15 was involved in regulating in ribosome biogenesis in human cells.

### RRP15 is involved in rRNA transcription

**Figure 4:**
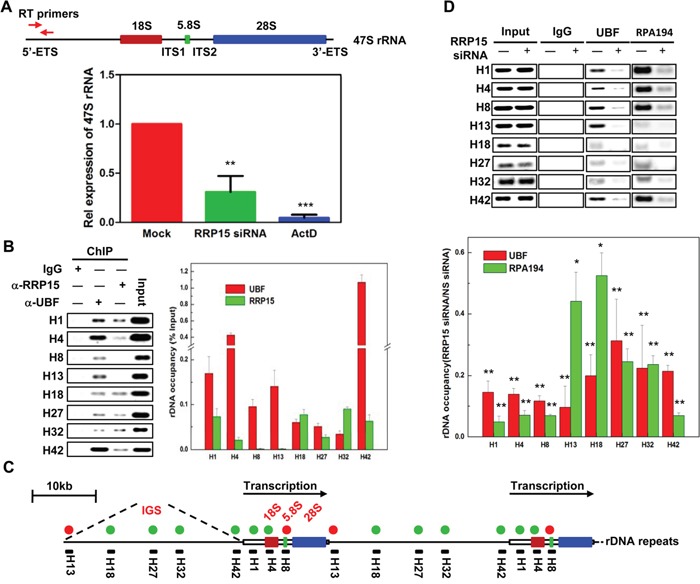
Regulation of 47S pre-rRNA transcription by RRP15 **A**. The effects of RRP15 on 47S pre-rRNA transcription. Total RNA from HeLa cells transfected with or without RRP15 siRNA for 48 h or treated with ActD for 12 h (positive control) was extracted and 47S pre-rRNA was amplified by real-time RT-PCR using specific primers as indicated in the diagram of 47S pre-rRNA. Histograms represented 47S pre-rRNA levels in HeLa cells treated with control, RRP15 siRNA and ActD. **B**. Determination of RRP15 binding sites in rDNA loci by ChIP analysis. HeLa cells were cross-linked and immunoprecipitated by IgG (negative control), α-UBF (positive control) or α-RRP15. DNA in IgG, α-UBF or α-RRP15 immunoprecipitates was amplified by indicated PCR primers and then separated by agrose gel electrophoresis. Histograms represented the percentages of DNA immunoprecipitated by α-RRP15 and α-UBF that were relative to input DNA. **C**. A schematic representation of human rDNA repeats with RRP15 binding sites. Green circle dots represented RRP15 binding sites and red circle dots represented RRP15 negative binding sites. Primer pairs (black solid bars) and their approximate positions relative to the transcription start are indicated. **D**. Perturbation of UBF and RPA194 association with rDNA loci by RRP15 depletion. HeLa cells transfected with or without RRP15 siRNA for 48 h were employed to perform ChIP analysis described in (B) using α-UBF and α-RPA194. Histograms represented the percentages of DNA immunoprecipitated by α-UBF and α-RPA194 that were relative to input DNA in cells transfected with or without RRP15 siRNA. *P<0.05 and **P<0.01.

In eukaryotic cells, ribosome biogenesis was initiated from 47S pre-rRNA transcription at rDNA repeats in the nucleolus. Subsequently, a series of rRNA processing was executed by exonuclease and endonuclease. The processed rRNAs together with the rRNA associated proteins then assembled into pre-40S and pre-60S ribosomal subunits (Supplementary Figure 7A and [1, 5, 9]). rRNA transcription at rDNA repeats and rRNA processing were considered as the critical rate-limiting steps for ribosome biogenesis in the nucleolus [34]. As RRP15 was required for nucleolar formation and ribosome biogenesis (Figure 1–3), we examined if, besides its role(s) in rRNA processing [35], RRP15 might also participate in regulating rRNA transcription. To this end, we made a pair of specific primers that amplified 5′-ETS of 47S pre-rRNA to monitor 47S pre-rRNA transcription using real-time reverse transcription-polymerase chain reaction (RT-PCR) (Figure 4A). Total RNA isolated from control cells, cells depleted of RRP15 or cells treated with ActD, was subjected to real-time RT-PCR amplification. The results showed that, in contrast to control cells, cells depleted of RRP15 displayed significant inhibition of 47S pre-rRNA synthesis whereas cells treated with ActD showed a drastic block of 47S pre-rRNA synthesis, indicating that RRP15 was involved in regulating rRNA transcription (Figure 4A).

To characterize the regulation of RRP15 on rRNA transcription in detail, we performed chromatin cross-linking and immunoprecipitation (ChIP) assays in HeLa cells. Cells were cross-linked with formaldehyde and cell lysates were then immunoprecipitated by α-RRP15 or α-UBF. DNA enrichment in the α-RRP15 or α-UBF-immunoprecipitates was amplified by PCR using sets of DNA primers spanning through the entire human rDNA repeats with a resolution of ~0.5-1 kb (Figure 4B-4C and [36]). ChIP analysis indicated that UBF bound to the entire rDNA repeats as previously reported whereas RRP15 only interacted with the transcription initiation region (H1 and H4) and the long intergenic spacers (IGS) (H18, H27 and H32) of the rDNA repeats (Figure 4B-4C) [13, 37]. As RRP15 was a ribosomal RNA processing protein, it was possibly that interaction of RRP15 with rDNA repeats was mediated by pre-rRNA. To determine this, we pre-treated formaldehyde cross-linked cell lysates with RNase I and then the lysates were immunoprecipitated by α-RRP15. ChIP analysis revealed that association of RRP15 with rDNA repeats was not affected by RNase I treatment, indicating that association of RRP15 with rDNA repeats was not mediated by pre-rRNA (Supplementary Figure 8).

To further determine how RRP15 binding to rDNA repeats affected rRNA transcription, we examined the possibility that RRP15 influenced Pol I transcriptional machinery. Despite many attempts, we were unable to detect RRP15 co-immunoprecipitation with UBF (negative data not shown). However, when we monitored UBF and RPA194 (a subunit of Pol I) with rDNA repeats in control cells or cells depleted of RRP15, we found that, in contrast to control cells, cells depleted of RRP15 displayed significantly decreased association UBF and RPA194 with rDNA repeats (Figure 4D). These results suggested that RRP15 binding to rDNA repeats participated in the regulation of rRNA transcription by affecting association of transcription machinery with rDNA repeats, perhaps by facilitating Pol I recruitment and/or stabilization on rDNA repeats.

### Regulation of cell proliferation and cell cycle progression by RRP15-dependent nucleolar formation/ribosome biogenesis in various human cells

**Figure 5:**
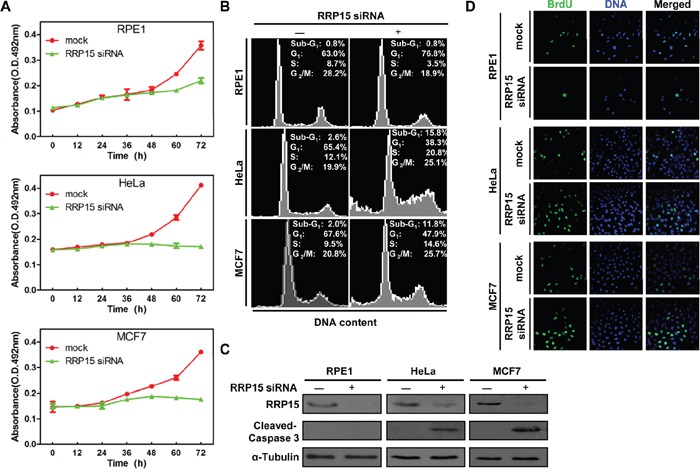
The effects of RRP15 ablation on cell proliferation and cell cycle progression in various human cells **A**. Inhibition of cell proliferation by RRP15 depletion in various human cells. RPE1 (up), HeLa (middle) and MCF7 cells (low) were transfected with or without RRP15 siRNA and cell proliferation was determined by MTT assay at indicated times. Results represent means ± standard deviations of five independent experiments. **B**. RPE1 (up), HeLa (middle) and MCF7 cells (low) were transfected with or without RRP15 siRNA for 48 h and cell cycle profiles were monitored by flow cytometry. Results are representative of three independent experiments. **C**. RPE1, HeLa and MCF7 cells were transfected with or without RRP15 siRNA for 48 h. The cell lysates were immunoblotted with α-RRP15, α-Caspase 3 and anti-α-Tubulin antibody. **D**. The effects of RRP15 depletion on BrdU (5-bromo-2′-deoxyuridine) incorporation in various human cells. Cells transfected with or without RRP15 siRNA for 48 h were labeled with 10 mM BrdU (1 h). The cells were fixed and immunostained with mouse α-BrdU. DNA was visualized by DAPI staining.

Given the fact that nucleolar formation/ribosome biogenesis is tightly linked with cell proliferation, cell cycle progression and checkpoint control [1, 15, 16], we next determined the effects of RRP15-dependent nucleolar formation/ribosome biogenesis on cell proliferation and cell cycle progression in human cells. Expression of RRP15 was ablated by RRP15 siRNA in non-transformed cell line, RPE1, and cancerous cell lines, HeLa or MCF7 (Supplementary Figure 9A). Cell proliferation analysis (MTT assay, see Materials and Methods) revealed that depletion of RRP15 in RPE1, HeLa or MCF7 cells resulted in inhibition of cell proliferation as compared with controls (Figure 5A). However, while non-transformed RPE1 cells depleted of RRP15 exhibited a 55% decrease in proliferation rate, cancerous HeLa or MCF7 cells depleted of RRP15 displayed complete inhibition of cell proliferation. Fluorescent-Activated Cell Sorting (FACS) analysis showed that, when compared with controls, depletion of RRP15 in RPE1 cells resulted in an increased accumulation of cells in G1 to G1/S phase whereas depletion of RRP15 in HeLa or MCF7 cells caused an accumulation of cells in S to G2/M phase (Figure 5B and Supplementary Figure 9B). In addition, depletion of RRP15 in HeLa or MCF7 cells also resulted in a marked accumulation of sub-G1 phase (cell death). Consistently, immunofluorescence or immunoblotting analysis showed that depletion of RRP15 in HeLa or MCF7 cells, but not RPE1 cells, induced apoptotic markers annexin V positive cell staining and the cleaved caspase 3 fragment (Figure 5C and Supplementary Figure 9C). Restoration of GFP-RRP15, but not GFP-RRP15^Δ228-232^, expression in HeLa cells depleted of RRP15 reduced annexin V positive cell staining (Supplementary Figure 5A and 9D). We monitored that depletion of RRP15 in HeLa or MCF7 cells caused cell death directly by time-lapse microscopy. In contrast to control cells that progressed through cell cycle and divided into two daughter cells normally (Supplementary Figure 10 dotted arrows and Supplementary Movie 1 and Movie 3), HeLa or MCF7 cells depleted of RRP15 displayed delayed cell cycle progression, ultimately undergoing cell death (Supplementary Figure 10 solid arrows and Supplementary Movie 2 and Movie 4).

We investigated how depletion of RRP15 resulted in G1-G1/S arrest in non-transformed RPE1 cells and S-G2/M phase block and cell death in cancerous HeLa or MCF7 cells. To this end, we monitored S-phase by BrdU incorporation in RPE1, HeLa or MCF7 cells treated with control or RRP15 siRNA. As shown in Figure 5D and Supplementary Figure 9E, when compared with controls, depletion of RRP15 expression in RPE1 cells resulted in significant reduction of BrdU incorporation whereas depletion of RRP15 in HeLa and MCF7 cells resulted in accumulation of BrdU incorporation. These results indicated that depletion of RRP15 in non-transformed RPE1 cells arrested cells in G1-G1/S phase and blocked S-phase entry. In contrast, depletion of RRP15 in cancerous HeLa or MCF7 cells did not inhibit cell cycle progression at G1-G1/S phase, resulting in that these cells entered S-phase, and, ultimately, caused cell death.

### RRP15 ablation induces nucleolar stress response in p53-proficient RPE1 cells but S-phase checkpoint response in p53-deficient HeLa or MCF7 cells

**Figure 6:**
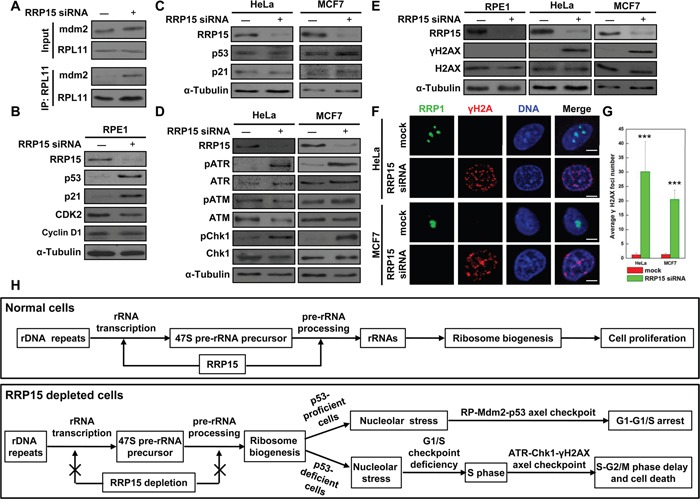
Induction of nucleolar stress response in p53-proficient RPE1 cells or S-phase checkpoint response in p53-deficient HeLa or MCF7 cells by RRP15 ablation **A**. RRP15 depletion in RPE1 cells enhanced Mdm2 interaction with RPL11. RPE1 cells were transfected with or without RRP15 siRNA for 48 h. Whole-cell lysates were subjected to immunoprecipitation and immunoblotting analyses. **B** and **C**. The effects of RRP15 depletion on expression of cell cycle related proteins in various human cells. RPE1, HeLa or MCF7 cells were transfected with or without RRP15 siRNA for 48 h. Whole cell lysates were immunoblotted with indicated antibodies. **D**. The effects of RRP15 depletion on the expression of DNA damage related proteins in various human cells. HeLa or MCF7 cells were transfected with or without RRP15 siRNA for 48 h. Whole cell lysates were immunoblotted with indicated antibodies. **E** and **F**. RRP15 depletion induced increases of γH2AX level and γH2AX foci formation in HeLa and MCF7 cells. RPE1, HeLa or MCF7 cells transfected with or without RRP15 siRNA for 48 h were immunoblotted or immunostained with indicated antibodies. Scale bars, 5 μm. **G**. Histograms represented average numbers of γH2AX foci detected in 20 cells in (F). ***P<0.001. **H**. The model of RRP15 involved in regulating ribosome biogenesis and cell proliferation (for detail, see text).

We determined the mechanism(s) by which depletion of RRP15 resulted in G1-G1/S phase arrest in RPE1 cells, and S-G2/M phase arrest and cell death in HeLa or MCF7 cells. Since depletion of RRP15 perturbed nucleolar formation/ribosome biogenesis, we reasoned that depletion of RRP15 in non-transformed p53-proficient RPE1 cells would activate RP-Mdm2-p53 axis nucleolar stress checkpoint response by dispersing RPL11 from the nucleolus into the nucleoplasm, increasing RPL11 interaction with Mdm2 and resulting in accumulation of p53 that arrested cells at G1-G1/S phase. Consistent with the speculation, immunoprecipitation and immunoblotting analyses showed that depletion of RRP15 in RPE1 cells caused significantly increased interaction of RPL11 with Mdm2 and accumulations of p53 and p53 downstream target, CDK inhibitor p21, when compared with controls (Figure 6A and 6B). In contrast, expression of cell cycle regulators, CDK2 and Cyclin D1, was not significantly altered in RPE1 cells depleted of RRP15 (Figure 6B). These results indicated that perturbation of RRP15-dependent nucleolar formation/ribosome biogenesis by RRP15 depletion activated RP-Mdm2-p53 axis nucleolar stress checkpoint response, thereby blocking non-transformed p53-proficient RPE1 cells at G1-G1/S phase.

However, as p53 function was defective in cancerous HeLa or MCF7 cells due to E6 oncoprotein expression in HeLa cells or Mdm2 oncoprotein overexpression in MCF7 cells [38–40], we found that, in contrast to RPE1 cells, depletion of RRP15 did not result in accumulations of p53 and p21 in p53-deficient HeLa or MCF7 cells (Figure 6C), although depletion of RRP15 perturbed nucleolar formation and induced nucleolar localized RPL11 dispersing from the nucleolus into the nucleoplasm in HeLa cells (Supplementary Figure 4A). Thus, RP-Mdm2-p53 axis nucleolar stress checkpoint response was not effectively activated in HeLa and MCF7 cells depleted of RRP15, resulting in that these cells entered S-phase (Figure 5B). However, as perturbation of RRP15-dependent nucleolar formation/ribosome biogenesis could affect protein synthesis (Figure 3 and Supplementary Figure 6A-6C) that might be required for further cell cycle progression (i.e. DNA replication), we speculated that these effects on HeLa or MCF7 cells depleted of RRP15 might activate ATM/ATR-Chk1/2- γH2AX axis DNA replication/damage checkpoint response in S-phase, delaying S-G2/M phase progression and ultimately causing cell death. To test the possibility, we examined DNA replication/damage checkpoint response in control and RRP15-depleted RPE1, HeLa or MCF7 cells. Immunoblotting analysis showed that depletion of RRP15 in HeLa or MCF7 cells, but not RPE1 cells, resulted in increased levels of phosphorylated ATR and Chk1, indicating that ATR/Chk1 DNA replication/damage checkpoint response was activated in HeLa or MCF7 cells depleted of RRP15 when compared with controls (Figure 6D and Supplementary Figure 11A). In addition, when compared with RPE1 cells, HeLa or MCF7 cells depleted of RRP15 also displayed significantly increased levels of ATR/Chk1 downstream target, γH2AX (phosphorylated H2AX) by immunoblotting analysis and γH2AX foci formation by immunofluorescence analysis (Figure 6E–6G). Inhibition of ATR/Chk1 DNA replication/damage checkpoint response in cells depleted of RRP15 by treatment of caffeine, a nonspecific inhibitor of PIKK, abrogated γH2AX induction (Supplementary Figure 11B-11D). Thus, these results, together with data obtained from FACS analysis and time-lapse microscopy (Figure 5B and Supplementary Figure 9 and 10), demonstrated that, in contrast to non-transformed p53-proficient RPE1 cells, perturbation of RRP15-dependent nucleolar formation/ribosome biogenesis by RRP15 depletion in p53-deficient HeLa or MCF7 cells did not effectively activate RP-Mdm2-p53 axis nucleolar stress checkpoint response to block cells at G1-G1/S. Instead, p53-deficient HeLa or MCF7 cells depleted of RRP15 entered S-phase with S-phase perturbation, activating ATR-Chk1- γH2AX axis DNA replication/damage checkpoint response that delayed S-G2/M phase progression and, ultimately, caused cell death.

## DISCUSSION

We investigated the functions of ribosomal RNA processing protein, RRP15, in human cells. Our results demonstrated that RRP15 was a nucleolar protein required for nucleolar formation. RRP15 participated in not only ribosome biogenesis but also rRNA transcription. Perturbation of RRP15-dependent nucleolar formation/ribosome biogenesis induced RP-Mdm2-p53 axis nucleolar stress checkpoint response and arrested cells at G1-G1/S in p53-proficient non-transformed RPE1 cells but activated ATR-Chk1- γH2AX axis DNA replication/damage checkpoint response and caused S-G2/M block and, ultimately, cell death in p53-deficient HeLa or MCF7 cells.

Previous studies showed that the budding yeast Rrp15p, a component of pre-60S subunits, was required for rRNA processing of 27SA2 at the C2 cleavage site and was involved in regulating the maturation of pre-60S ribosomal subunit [32]. Although depletion of Rrp15p inhibited cell proliferation, large-scale affinity purification analyses by mass-spectrometry did not define the precise maturation stage(s) of the pre-ribosomal particles containing Rrp15p. Recently, based on the nucleolar proteomics obtained from various organisms, Lionel Tafforeau et al. selected 625 candidates localized at the nucleolus and examined these proteins involved in pre-rRNA processing and ribosome biogenesis. They found that RRP15 was involved in regulating the maturation of pre-40S and pre-60S rRNA intermediates. Depletion of RRP15 resulted in accumulation of 41S rRNA intermediate and reductions of 21S and 12S rRNA intermediates, indicating that RRP15 was required for early pre-rRNA processing steps that was critical for both pre-40S and pre-60S ribosome biogenesis [35]. Consistent with these findings, our results revealed that RRP15 was components of both pre-40S and pre-60S subunits, thus indicating that RRP15 participated in the control of pre-40S and pre-60S ribosome biogenesis.

Several studies demonstrated that rDNA active transcription played a crucial role for nucleolar formation [41, 42]. As RRP15 was required for nucleolar formation, depletion of RRP15 inhibited 47S pre-rRNA transcription and reduced association of Pol I with rDNA repeats. These results, together with the role of RRP15 in pre-rRNA processing [35], suggested that RRP15 could be a multifunctional factor involved in regulating ribosome biogenesis at different stages (Supplementary Figure 7B). Nucleolar proteins, such as nucleolin and nucleophosmin, were shown to have multiple roles in regulating ribosome biogenesis at different stages. Nucleolin, a histone chaperone with a FACT-like (FAcilitates Chromatin Transcription) activity, was involved in not only rRNA transcription but also rRNA processing and ribosomal assembly. Depletion of nucleolin perturbed nucleolar organization, inhibited rRNA transcription by affecting chromatin remodeling and histone dynamics and blocked early steps of rRNA processing in the 5′ ETS [11–13, 43]. Nucleophosmin was involved in regulating rRNA transcription by recruiting the transcriptional factor c-Myc to rDNA repeats [44]. However, nucleophosmin also functioned as an endoribonuclease whose activity played an important role in pre-rRNA processing in the ITS2 [45]. Hence, as a coiled-coil protein, RRP15 could interact with crucial nucleolar proteins to assemble multiple complexes required for regulating rRNA transcription and/or rRNA processing/ribosomal assembly in the nucleolus (Figure 6H). NOP16, a potential RRP15 associated protein identified by bioinformatic studies, was reported to have important roles in governing rRNA transcription and regulating ribosome assembly [46].

A mounting body of evidence demonstrated that perturbations of nucleolar function/ribosome biogenesis generated nucleolar stress that inhibited cell cycle progression and activated the canonical RP-Mdm2-p53 axis checkpoint response for abnormal cell growth and transformation protection [12, 21, 24, 47]. Consistent with these studies, our results showed that perturbation of RRP15-dependent nucleolar formation and ribosome biogenesis that induced nucleolar stress activated RP-Mdm2-p53 axis checkpoint response and arrested cells at G1-G1/S in p53-proficient non-transformed cells. However, our results further revealed that RRP15-dependent nucleolar stress could not activate RP-Mdm2-p53 axis checkpoint response and arrest cells at G1-G1/S in p53-deficient tumor cells. Instead, these p53-deficient tumor cells with RRP15-dependent nucleolar stress entered S-phase with S-phase perturbation, activating ATR-Chk1- γH2AX axis DNA replication/damage checkpoint response that delayed S-G2/M phase progression and ultimately caused cell death (Figure 6H). Perturbation of nucleolar formation and ribosome biogenesis by depletion/mutation of critical nucleolar proteins and/or treatment with RNA transcription inhibitor ActD was reported to cause cell death in p53-deficient tumor cells [24, 27–29, 48]. In addition, previous studies demonstrated that varieties of endo- and/or extra-cellular stresses could affect nucleolar formation and/or ribosome biogenesis, which, in turn, turn on nucleolar-stress dependent checkpoint responses. For instance, in response of DNA damage (UV, IR or chemotherapy drug treatment), reactive oxygen species (ROS) induction or nutrient deprivation, nucleolar formation and/or function would be impaired, resulting in abnormalities of nucleolar formation/function that would stabilize p53 and induce nucleolar-stress dependent checkpoint responses [21, 49–51]. It could be possible that nucleolar stress checkpoint response and other cell cycle checkpoint responses including DNA damage checkpoint response could sever as a regulatory control loop(s) to monitor cell cycle progression in eukaryotic cells.

In summary, we investigated ribosomal RNA processing protein RRP15 involved in regulating nucleolar formation, ribosome biogenesis, cell proliferation, cell cycle progression and checkpoint control in human non-transformed and cancer cells. Our study provides new insights into how perturbation of RRP15-dependent nucleolar formation/ribosome biogenesis caused cell death in p53-deficient tumor cells. Thus, the selective checkpoint response, cell cycle inhibition and cytotoxicity induced by RRP15-dependent nucleolar stress in p53-proficient non-transformed cells and p53-deficient tumor cells suggest that RRP15 might be a potential target for cancer therapy.

## MATERIALS AND METHODS

### Cell culture, transfection and drug treatment

Human cervical carcinomas HeLa cells, human breast adenocarcinoma MCF7 cells and RPE1 (hTERT-RPE1) cells were purchased from ATCC. The validation of the identity of HeLa, MCF7 and RPE1 cells was determined by Tianjin Weikai Bioeng LTD (China, Tianjin). HeLa and MCF7 cells were cultured in DMEM (HyClone) supplemented with 10% fetal bovine serum (FBS) (HyClone). RPE1 cells were cultured in DMEM: F-12 (1:1) (HyClone) containing 10% FBS. All cells were cultured at 37°C in 5% CO_2_. Plasmid/siRNA transfection was conducted with Lipofectamine 3000 and/or RNAiMAX Reagent (Life Technologies Inc) according to manufacturer's protocol. In brief, HeLa, MCF7 and RPE1 cells were plated in 96-well plates (3000 cells/well), 24-well plates (2×10^4^ cells/well) or 6-well plates (1×10^5^ cells/well) for 16 h at 37°C before transfection. Then, cells were incubated with indicated plasmids/siRNAs plus transfection reagent mixture in medium for 24 h and then changed into fresh medium. Cycloheximide (CHX) (Aladdin) and Actinomycin D (ActD) (Byotime) were used for cell treatment at final concentration of 10 μM CHX and 5 nM ActD at indicated times as previously described [52].

### Preribosome and polysome preparation

Nuclear extracts were fractionated and preribosome preparation was performed as described previously [31] with minor modifications. In brief, HeLa cells were swollen in ice-cold hypotonic lysis buffer (10 mM Tris [pH 7.4], 10 mM KCl, 2 mM MgCl_2_, 0.05% Triton X-100, 1 mM EGTA, 1 mM DTT, 40 mg/ml of phenylmethylsulfonyl fluoride, and 10 mg/ml of protease inhibitor cocktail). The nuclei pellet was collected by centrifugation at 500g for 5 min. The nuclear lysate was extracted in extraction buffer (25 mM Tris (pH 7.5), 100 mM KCl, 1 mM DTT, 2 mM EDTA, 0.1% NP-40, 1 mM NaF, 40 mg/ml of phenylmethylsulfonyl fluoride, 10 mg/ml of protease inhibitor cocktail and 0.1U/ml of RNasin (Promega)) and sonicated. The nuclear lysate was overlaid on 10 to 30% (wt/wt) sucrose gradients in preribosome buffer (25 mM Tris (pH 7.5), 100 mM KCl, 1 mM DTT and 2 mM EDTA) and centrifuged at 36,000 rpm for 3 h at 4°C in a Beckman SW41Ti rotor. The gradients were collected downward and the absorbance of each fraction was measured at 260 nm using a spectrophotometer.

Polysome preparation was performed as described previously [31] with minor modifications. In brief, HeLa cells were collected and suspended in lysis buffer (20 mM Tris-HCl [pH 7.4], 130 mM KCl, 10 mM MgCl_2_, 2.5 mM dithiothreitol, 0.5% NP-40, 0.5% sodium deoxycholate, 10 μg/ml CHX, 0.2 mg/ml heparin and 200U/ml RNase inhibitor) and incubated on ice for 15 min. Lysates were centrifuged at 8,000g for 15 min and supernatants were collected. Supernatants were layered over 10 to 45% (wt/wt) sucrose density gradients in polysome buffer (10 mM Tris-HCl [pH 7.4], 60 mM KCl, 10 mM MgCl_2_, 1 mM dithiothreitol and 0.1 mg/ml heparin) and centrifuged at 36,000 rpm for 3 h at 4°C in a Beckman SW41Ti rotor. The gradients were collected downward and the absorbance of each fraction was measured at 260 nm using a spectrophotometer.

### Chromatin cross-linking and immunoprecipitation (ChIP)

ChIP analysis was performed using the ChIP Assay Kit (upstate) according to manufacturer's protocol. The chromatin solution was immunoprecipitated with indicated antibodies. The immunoprecipitates were amplified by PCR using sets of DNA primers spanning through the entire human rDNA repeats with a resolution of ~0.5-1 kb as described previously [36]. PCR products were separated by agrose gel electrophoresis and densitometries of electrophoretic bands were quantitated by using Scion Image software.

### Cell proliferation (MTT) assay, cell cycle analysis and BrdU incorporation assay

For cell proliferation (MTT) assay, HeLa, MCF7 or RPE1 cells were plated in 96-well plates (3000 cells/well) for 16 h. Cells were then transfected with or without RRP15 siRNA using RNAiMAX Transfection Reagent for 24 h. After transfection, cells were changed into fresh medium and determined by MTT (3-(4,5-dimethylthiazol-2-yl)-2,5-diphenyltetrazolium bromide) analysis at indicated times as described previously [53].

For cell cycle analysis, HeLa, MCF7 or RPE1 were fixed in 70% ethanol/30% phosphate-buffered saline (PBS) for 1 h at -20°C. After fixation, cells were washed once with PBS, resuspended, and incubated in propidium iodide (PI) buffer (60 μg/ml PI and 0.1 mg/ml RNase A) for 45 min at room temperature. Flow cytometry was conducted on at least 5,000 cells per condition using a FACSort and CellQuest version 3.3 (BD Biosciences). Cell cycle profiles were processed and analyzed using FlowJo version 6.4.7 (Tree Star, Ashland, OR).

BrdU incorporation was performed as described previously [23]. Cells were fixed and immunostained with mouse α-BrdU. The percentages of BrdU positive cells were scored (>1000 cells) using a fluorescence microscope.

Extended materials and experimental procedures can be found in Supplementary Materials and Methods.

## SUPPLEMENTARY MATERIALS AND METHODS, REFERENCES, FIGURES AND MOVIES










