# Supplementary material for: The roles of RRP15 in nucleolar formation, ribosome biogenesis and checkpoint control in human cells
Source: Oncotarget. 2017 Jan 14;8(8):13240–52. doi: 10.18632/oncotarget.14658 (PMC5355092; doi:10.18632/oncotarget.14658)
Supplement: Supplementary file 1 [file oncotarget-08-13240-s001.pdf]

The roles of RRP15 in nucleolar formation, ribosome biogenesis and checkpoint control in human cells

SUPPLEMENTARY MATERIALS AND METHODS

Plasmids, siRNAs and antibodies

The full-length coding region of RRP15 cDNA and its point mutation or deletion mutants were generated by PCR and subcloned into EcoRI–SalI sites of the mammalian expression vector pEGFP-C2. All constructs were fully sequenced. siRNA specific targeting to RRP15 (5'-AAATGGTAACTGGAGCCGTA-3') were synthesized by Genepharma (Shanghai, China). esiRNAs specific targeting to 3'-UTR of RRP15 were generated as previously described [1]. Polyclonal rabbit α-RRP15 were generated against bacterially expressed, purified His-tagged RRP15 fusion protein. Mouse α-UBF (#sc-13125), mouse α-p53 (#sc-126), rabbit α-p21 (#sc-397), mouse α-nucleolin (#sc-8031) and mouse α-Mdm2 (#sc-965) were purchased from Santa Cruz Biotechnology. Mouse α-fibrillarin (#ab4566) was purchased from Abcam. Mouse α-BrdU (#5292), mouse α-RPS6 (#2317) and rabbit α-CDK2 (#ab47518) were purchased from Cell Signaling Technology. Mouse α-RPL11 (#37-3000) was purchased from Life Technologies Inc. Mouse α-Bip (#27033) was purchased from signalway antibody. Mouse α-γH2AX (05-636) was purchased from Merk Millipore. Rabbit α-H2AX (#3522-1), rabbit α-ATR (#2790), rabbit α-pATR (S428) (#2853), rabbit α-ATM (#1459-1), rabbit α-pATM (T1981) (#2152-1), rabbit α-Chk1 (#2865-1), rabbit α-pChk1 (S345) (#S0660), and rabbit α-cyclin D1 (#2261-1) were purchased from Epitomics. Mouse anti-α-tubulin (T5168) antibody was purchased from Sigma-Aldrich. All secondary antibodies were obtained from Life Technologies Inc.

Immunoblotting, immunoprecipitation, and immunofluorescence analyses

Cells or cells transfected with indicated plasmid and/or siRNA or treated with indicated drug were harvested or fixed for immunoblotting, immunoprecipitation or immunofluorescence analysis as previously described [2]. In brief, for immunoblotting, cells harvested and lysed in 1% Nonidet P-40 buffer [3]. Cell lysates with equal amounts of total protein were subjected to SDS-PAGE, transferred to PVDF membrane and then immunoblotted with corresponding antibodies. For immunoprecipitation, cell lysates were immunoprecipitated with indicated antibodies. The immunoprecipitates were subjected to immunoblotting with indicated antibodies. For immunofluorescence analysis, cells grown on glass coverslips were fixed and immunostained with indicated antibodies. Cells were photographed using a NIKON fluorescence microscope. Mean intensity was measured in a 0.19-μm^2 area at nucleolus, nucleoplasm, and cytoplasm in each of 20 cells using Image-Pro Plus 7.0 (Media Cybernetics). Pearson's correlation coefficients (R value) were calculated using Image-Pro Plus 7.0 [4].

RNA isolation and real-time RT-PCR

Total RNA was isolated using the Trizol reagent (Invitrogen) following manufacturer's instructions. One microgram RNA was used for cDNA synthesis using a reverse transcriptase reaction kit (Promega) and quantitative real-time PCR was performed on an ABI Prism 7000 Sequence Detection System (Applied Biosystems), using SYBR Green (TIANGEN BIOTECH) as a dsDNA-specific fluorescent dye. β-actin was used for standardizing 47S rRNA level. Amplification primers were 5'-TCGAGCGTTCGCGTTCAG-3' and 5'-GAGTGAGACGAGACGAGACGC-3' for 47S rRNA, 5'-GGTAACTGGAGCCGTAG-3' and 5'-GGACTTTAGCCATAGCAT-3' for RRP15, and 5'-TCGTGCGTGACATTAAGGAG-3' and 5'-ATGCCAGGGTACATGGTGGT-3' for β-actin. Data were analyzed by using the $2^{-\Delta\Delta Ct}$ method [5]. All results represent means ± standard deviations of three independent experiments.

Time-lapse microscopy

Cells grown on 35-mm glass-bottom microwell dishes (MatTek, Ashland, MA) were transfected with 0.2 µg of pGPU6/GFP-NC shRNA (Control) and pGPU6/GFP-RRP15 shRNA (RRP15 siRNA) plasmids using Lipofectamine 3000 according to manufacturer's protocol. Twenty-four hours after transfection, dishes were then covered with mineral oil (Sigma) and transferred to a heated stage (37°C) on a NIKON Eclipse TS100 microscope (NIKON). Phase-contrast and fluorescence images of live cells were collected at 2 min intervals for 24 h and processed using the AR 4.0 software (NIKON).

Statistical analysis

Student's test was used to calculate the statistical significance of the experimental data. The level of significance was set as *P<0.05, **P<0.01 and ***P<0.001.

REFERENCES

1. Zhu C, Zhao J, Bibikova M, Leverson JD, Bossy-Wetzel E, Fan JB, Abraham RT, Jiang W. Functional analysis of human microtubule-based motor proteins, the kinesins and dyneins, in mitosis/cytokinesis using RNA interference. Mol Biol Cell. 2005; 16:3187-3199.
2. Zhu C, Jiang W. Cell cycle-dependent translocation of PRC1 on the spindle by Kif4 is essential for midzone formation and cytokinesis. Proc Natl Acad Sci U S A. 2005; 102:343-348.
3. Jiang W, Jimenez G, Wells NJ, Hope TJ, Wahl GM, Hunter T, Fukunaga R. PRC1: a human mitotic spindle-associated CDK substrate protein required for cytokinesis. Mol Cell. 1998; 2:877-885.
4. Zinchuk V, Zinchuk O, Okada T. Quantitative colocalization analysis of multicolor confocal immunofluorescence microscopy images: pushing pixels to explore biological phenomena. Acta Histochem Cytochem. 2007; 40:101-111.
5. Livak KJ, Schmittgen TD. Analysis of relative gene expression data using real-time quantitative PCR and the 2(-Delta Delta C(T)) Method. Methods. 2001; 25:402-408.

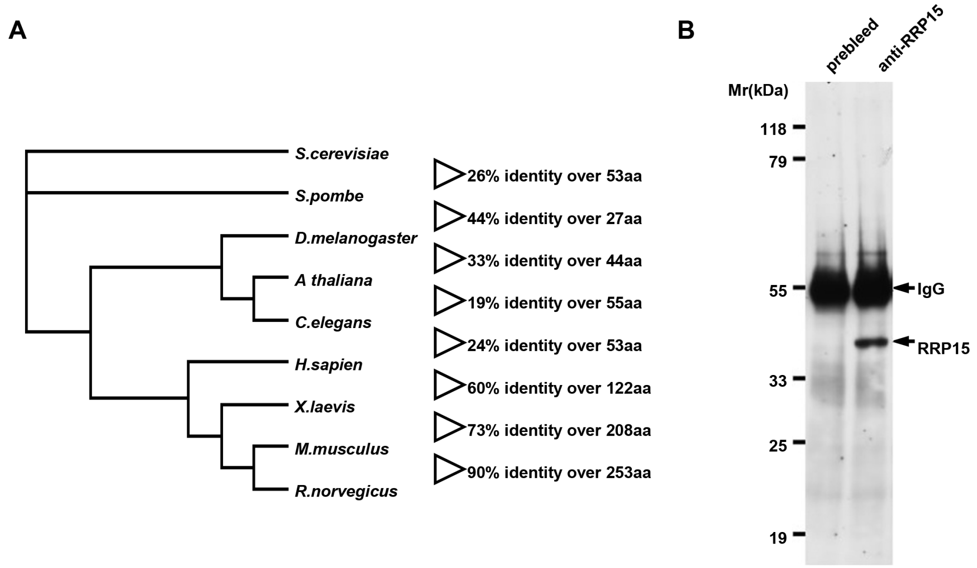

Supplementary Figure 1: The phylogenetic tree of RRP15 in eukaryotes and verification of α-RRP15. A. Shown is the phylogenetic tree of RRP15 proteins in various eukaryotic organisms. **B.** HeLa cell lysates were immunoprecipitated by rabbit anti-RRP15 sera or pre-bleed sera. The immunoprecipitates were separated by SDS-PAGE and immunoblotted with anti-RRP15 sera. Arrows indicated IgG and 45kDa RRP15 protein.

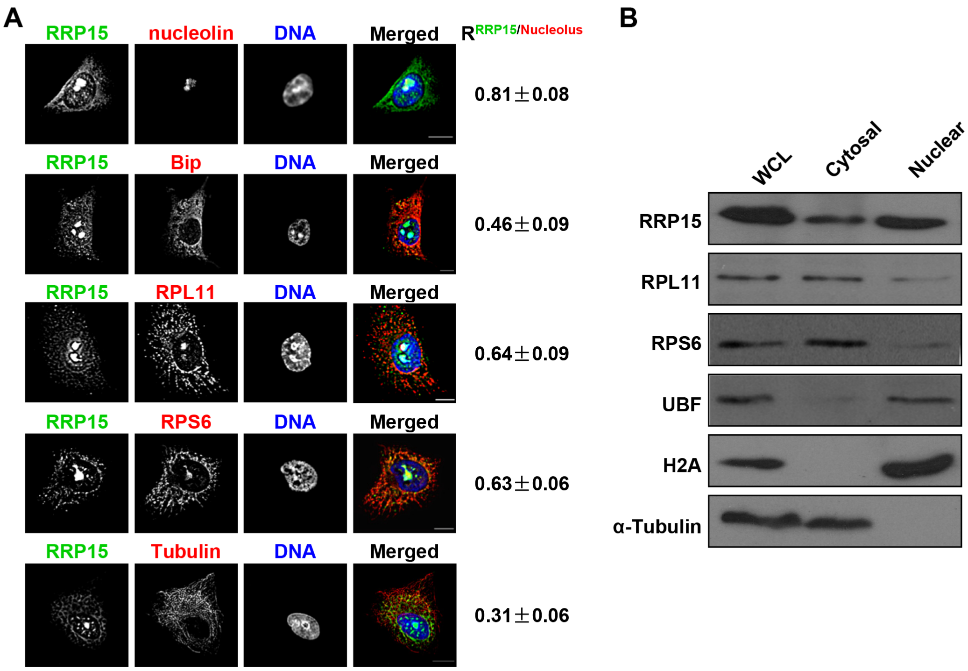

Supplementary Figure 2: The nucleolar and cytoplasmic localization of RRP15. A. HeLa cells were immunostained with rabbit α-RRP15 and mouse α-nucleolin, α-Bip, α-RPL11, α-RPS6 or anti-α-tubulin antibody. DNA was visualized by DAPI staining. R values were obtained as described (see supplementary Materials and Methods). Scale bars, 5 µm. **B.** Nuclear or cytoplasmic lysates from HeLa cells were extracted and subjected to immunoblotting by indicated antibodies.

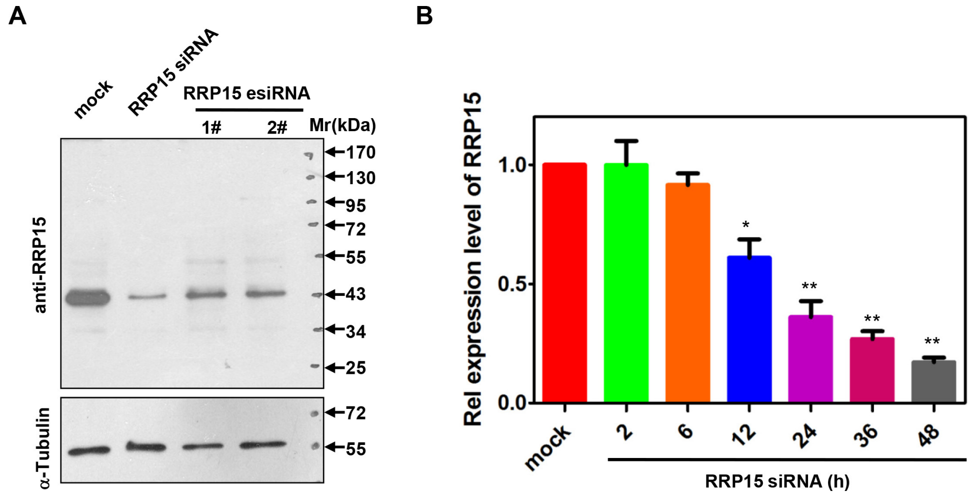

Supplementary Figure 3: Ablation of RRP15 mRNA and protein by RRP15 siRNA or RRP15 esiRNA. A. HeLa cells were transfected with control, RRP15 siRNA or RRP15 esiRNAs for 48 h. Cell lysates were separated by SDS-PAGE and immunoblotted with α- RRP15 and anti-α-Tubulin antibody. **B.** HeLa cells were transfected with control or RRP15 siRNA as described in (A). Transfected cells were collected at indicated times and total RNA was extracted. Expression of RRP15 mRNA was determined by real-time RT-PCR. *P<0.05 and **P<0.01.

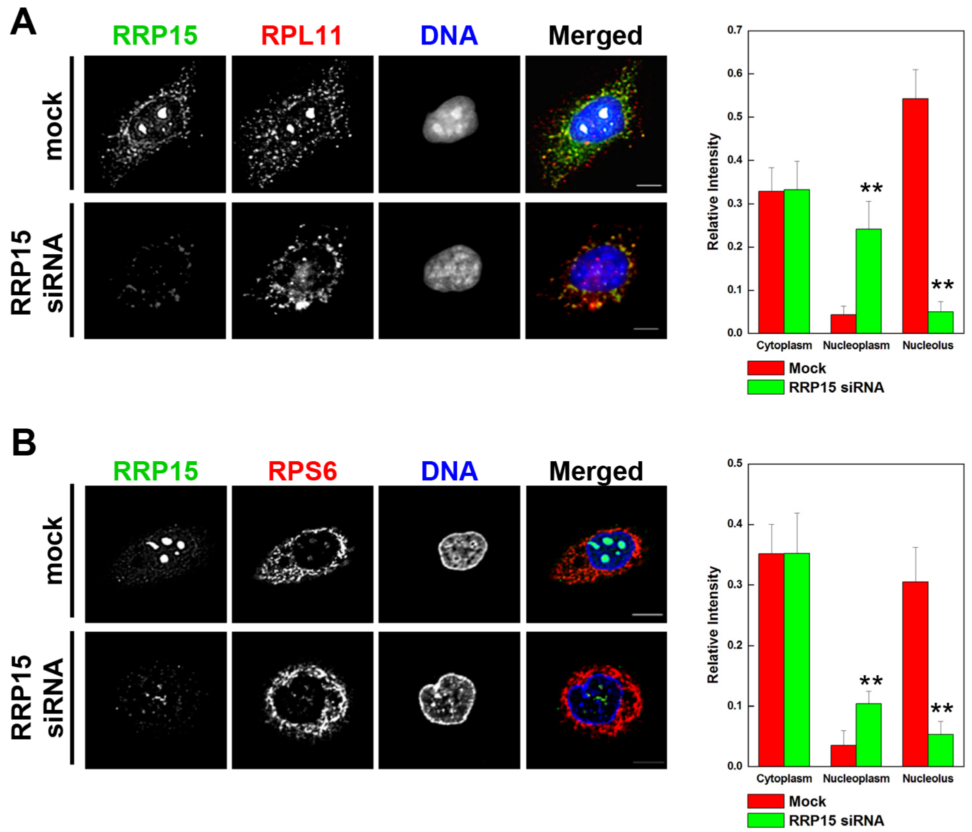

Supplementary Figure 4: The effects of RRP15 depletion on RPS6 and RPL11 localization. HeLa cells grown on coverslips were transfected with or without RRP15 siRNA for 48 h, fixed and immunostained with α-RRP15 and α-RPL11 (A) or α-RRP15 and α-RPS6 (B) DNA was visualized by DAPI staining. Scale bars, 5 μm. Intensities of RPL11 or RPS6 staining in the cytoplasm, nucleoplasm and nucleolus in 20 cells were determined by Image-Pro Plus 7.0. ** P<0.01.

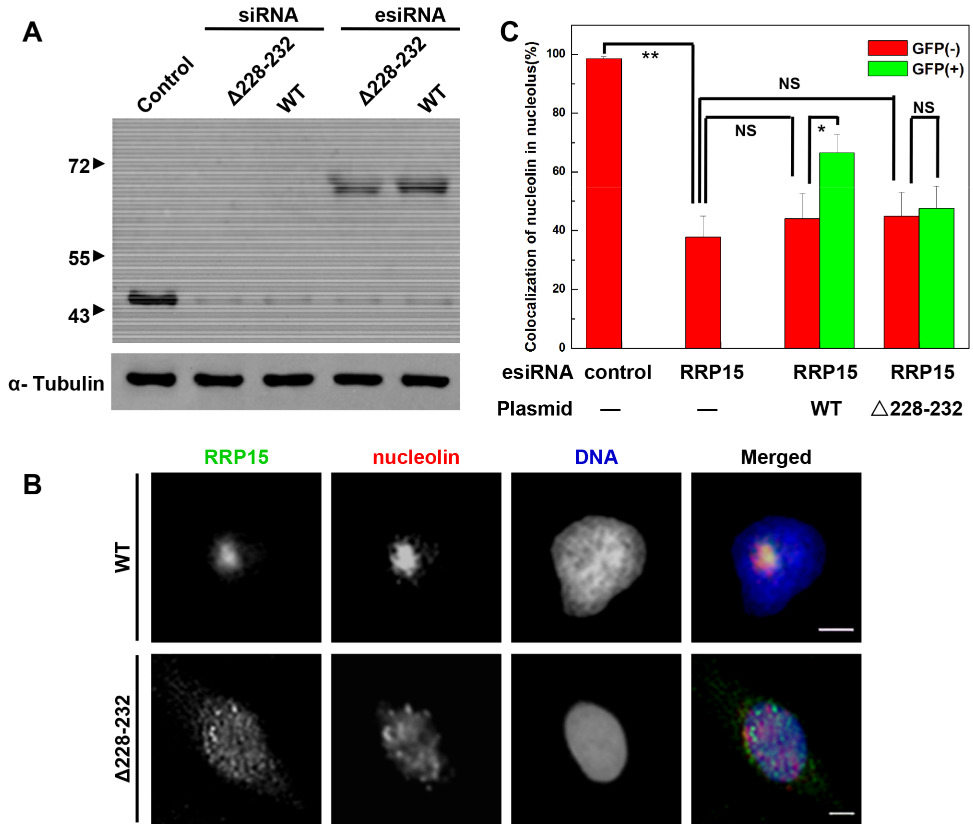

Supplementary Figure 5: Restoration of the nucleoli by expression of EGFP-RRP15 or EGFP-RRP15$^{\Delta 228\text{-}232}$ in cells depleted of RRP15. A. HeLa cells were transfected with pEGFP-RRP15 or pEGFP-RRP15$^{\Delta 228\text{-}232}$. Twenty-four hours later, cells were transfected with RRP15 siRNA or esiRNA. Two days after siRNA/esiRNA transfection, cells were lysed and immunoblotted with α-RRP15 or anti-α-tubulin antibody (Control). **B.** HeLa cells grown on coverslips were transfected with plasmids and esiRNAs as described in (A). Cells were fixed and immunostained with mouse α-nucleolin. DNA was visualized by DAPI staining. Scale bars, 5 µm. **C.** Histograms represented the percentage of cells with nucleolin localization in the nucleolus (>300 cells) in (B). **P<0.01.

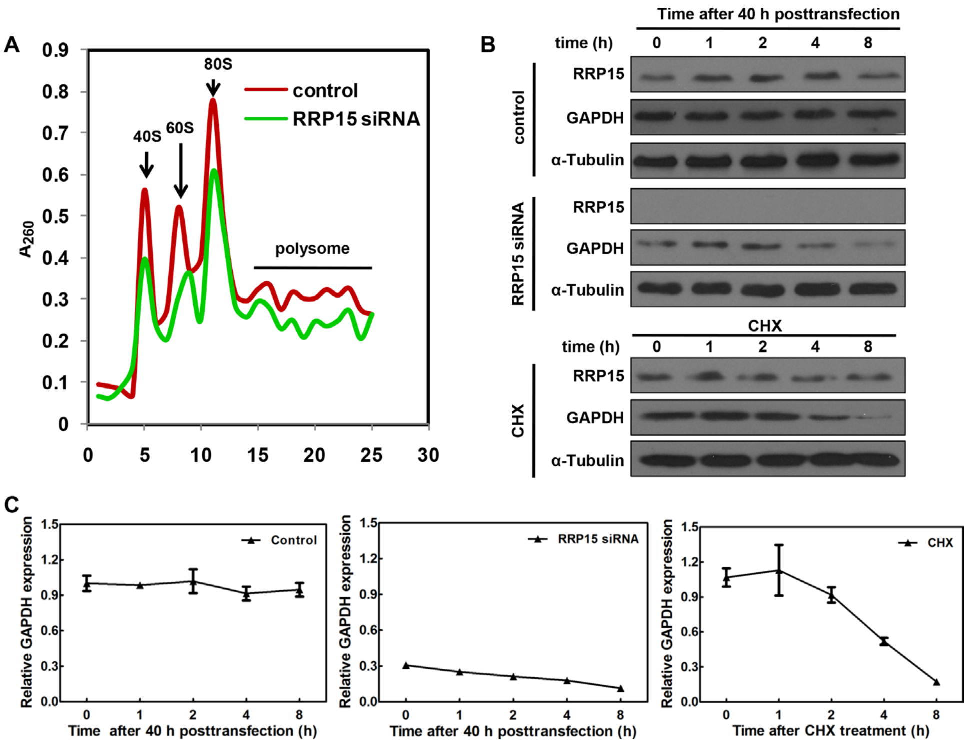

Supplementary Figure 6: The effects of RRP15 on ribosome biogenesis and protein synthesis. A. Ribosomal subunits and polysomes in the cytoplasm impaired by RRP15 depletion. HeLa cells were transfected with or without RRP15 siRNA for 48 h. Ribosomal subunits and polysomes were purified and profiled as described (see Materials and Methods). The positions of ribosomal subunits and polysome were indicated. **B.** HeLa cells were transfected with or without RRP15 siRNA for 40 h or HeLa cells treated with CHX were collected at indicated times and immunoblotted with indicated antibodies. **C.** The density of GAPDH in each panel in (B) was quantitated against the level of α-tubulin and the graphs were plotted.

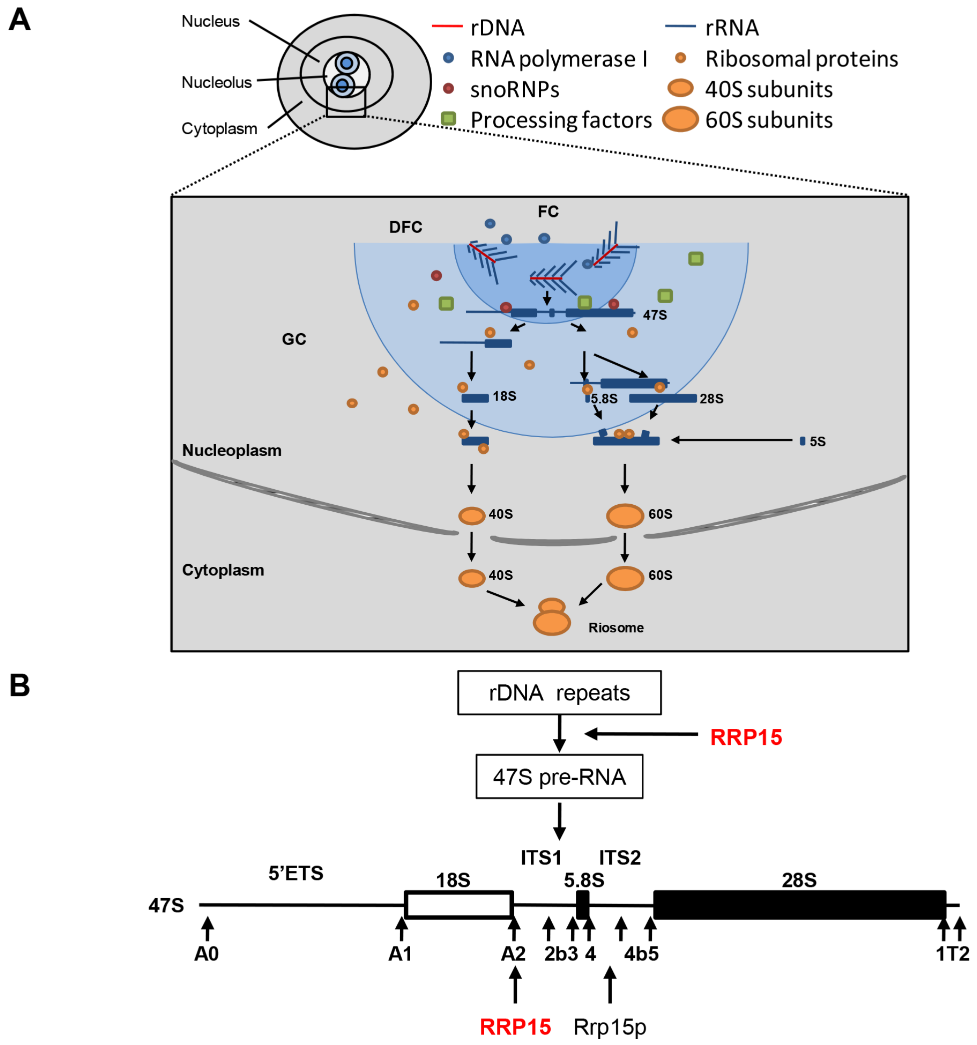

Supplementary Figure 7: A schematic diagram of ribosome biogenesis in mammalian cells and a schematic representation of RRP15 or Rrp15p in regulating ribosome biogenesis in mammalian cells or yeast. A. Shown is a schematic diagram of ribosome biogenesis in mammalian cells. **B.** Shown is a schematic representation of RRP15 or Rrp15p regulating ribosome biogenesis in mammalian cells or yeast.

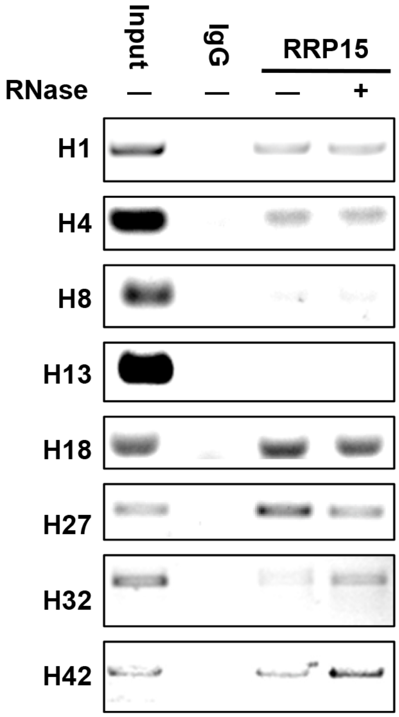

Supplementary Figure 8: Interaction of RRP15 with rDNA repeats is not mediated by pre-rRNA. Formaldehyde cross-linked HeLa cells were lysed and treated with 10 μg/ml RNase I as indicated. Cell lysates were immunoprecipitated by IgG or α-RRP15. DNA in IgG or α-RRP15 immunoprecipitates was amplified by indicated PCR primers, separated by agrose gel electrophoresis and visualized by ethidium bromide (EB) staining.

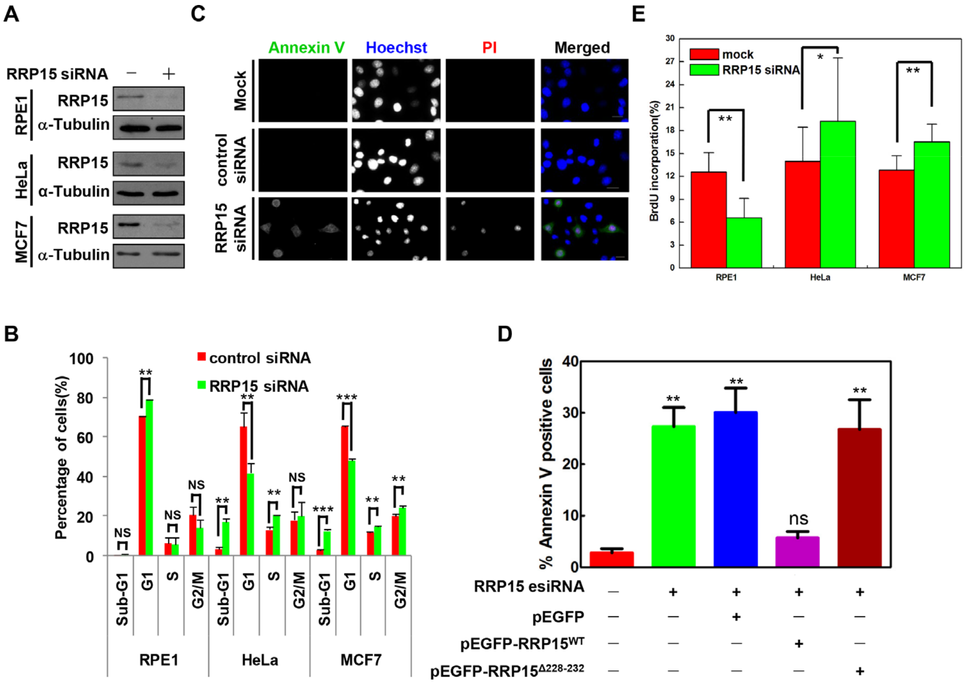

Supplementary Figure 9: The effects of RRP15 depletion on cell cycle progression, cell death and BrdU incorporation in various human cells. A. RPE1, HeLa and MCF7 cells were transfected with or without RRP15 siRNA for 48 h. Cell lysates were immunoblotted with α-RRP15 and anti-α-Tubulin antibody. **B.** Histograms represented the cell cycle distribution of various human cells after 48 h of RRP15 siRNA transfection. Results are representative of three independent experiments. **C.** HeLa cells grown on coverslips were transfected with or without RRP15 siRNA for 48 h. The cells were fixed and stained with Annexin V and PI. DNA was visualized by Hoechest33342 staining. Scale bars, 10 µm. **D.** HeLa cells were transfected with pEGFP, pEGFP-RRP15^{WT} or pEGFP-RRP15$^{\Delta228\text{-}232}$. Twenty-four hours later, cells were transfected with RRP15 esiRNA. Two days after esiRNA transfection, cells fixed and labeled with Annexin V and PI. The percentage of annexin V positive cells were calculated. More than 300 cells were counted in each experiment. **E.** Histograms represented the percentage of BrdU incorporation in indicated human cells depleted of RRP15. *P<0.05, **P<0.01 and***P<0.001.

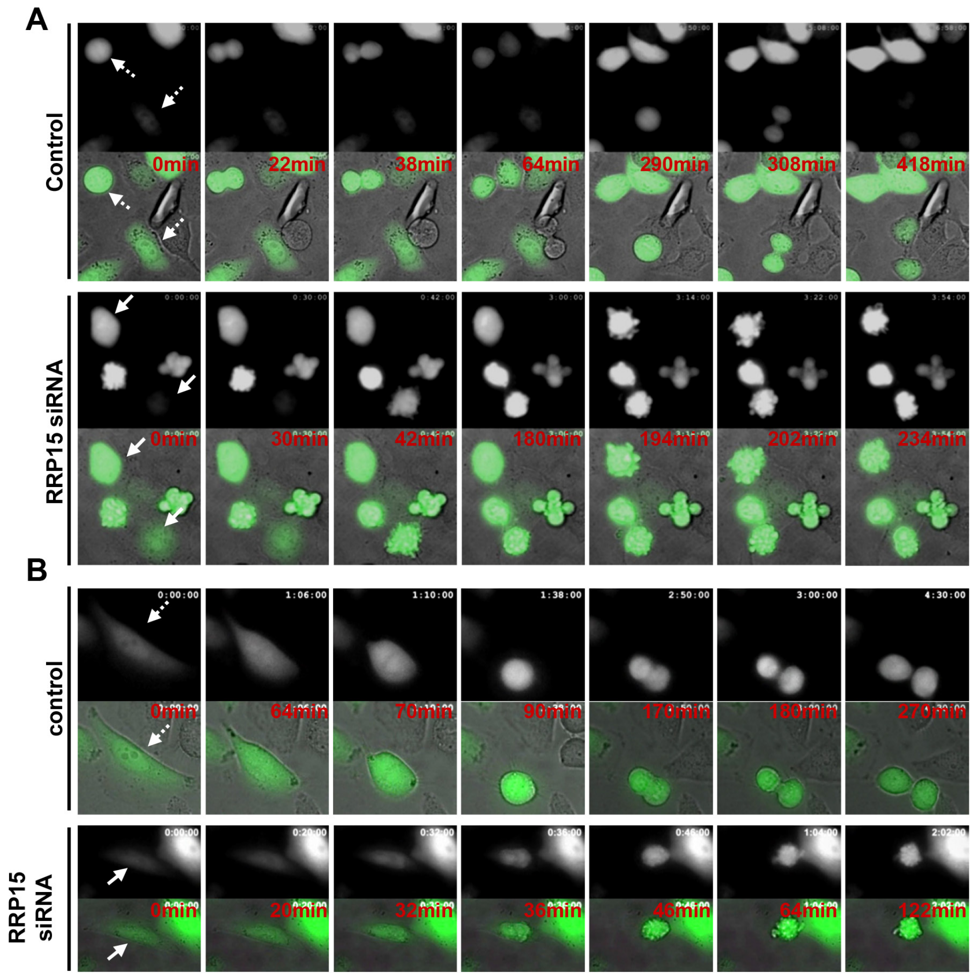

Supplementary Figure 10: Time-lapse microscopy of HeLa and MCF7 cells depleted of RRP15. HeLa (A) and MCF7 (B) Cells grown on glass-bottom microwell dishes were transfected with pGPU6/GFP-NC shRNA (Control) and pGPU6/GFP-RRP15 shRNA (RRP15 siRNA) plasmids for 24 h. Time-lapse images were collected every 2 min for 24 h. Representative images of the movies are shown (EGFP, pseudocolored green). Dotted arrows and solid arrows represented divided and dead cells, respectively. For details, see Supplementary Movies 1-4.

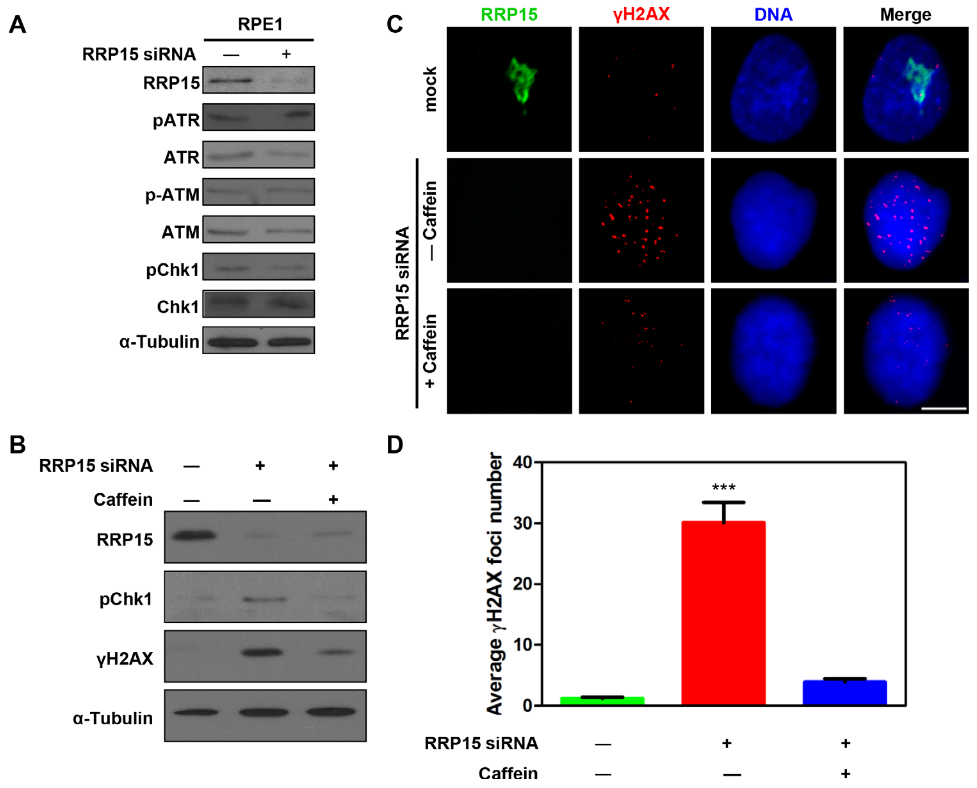

Supplementary Figure 11: The effects of RRP15 depletion on expression of DNA damage related proteins and γH2AX foci formation in human cells. A. RPE1 cells were transfected with or without RRP15 siRNA for 48 h. The whole cell lysates were immunoblotted with indicated antibodies. **B-C.** HeLa cells were transfected with or without RRP15 siRNA. After 36 h later, Cells were treated with or without 2.5 mM caffeine for 12 h. The cells were immunoblotted (B) or immunostained (C) with indicated antibodies. Scale bars, 10 μm. **D.** Histograms represented average numbers of γH2AX foci detected in 20 cells in (C). ***P<0.001.

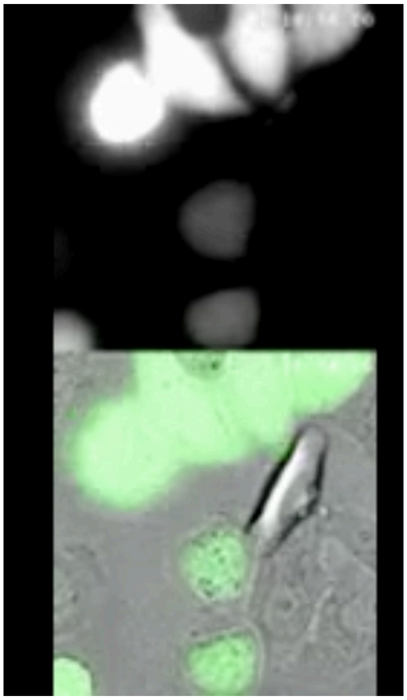

Supplementary Movie 1: Related to Figure 10A. HeLa cells transfected with control shRNA were imaged using microscopy.

See Supplementary Movie 1

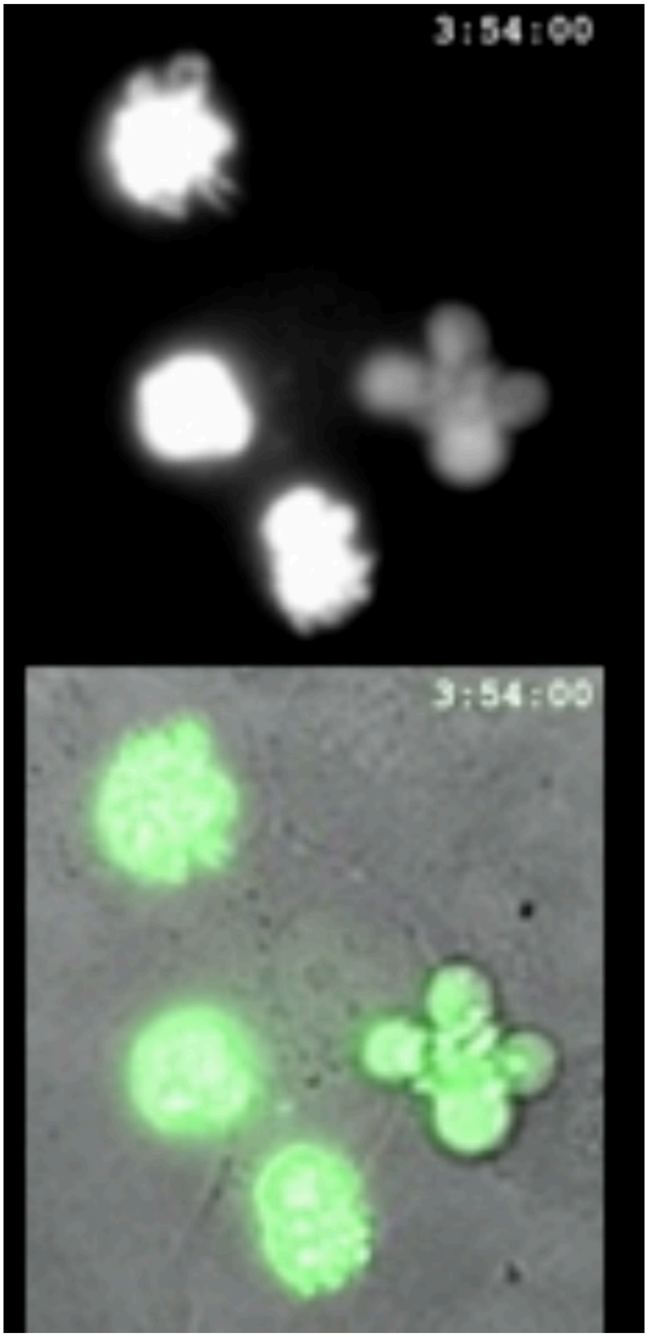

Supplementary Movie 2: Related to Figure 10A. HeLa cells transfected with RRP15 shRNA were imaged using microscopy.

See Supplementary Movie 2

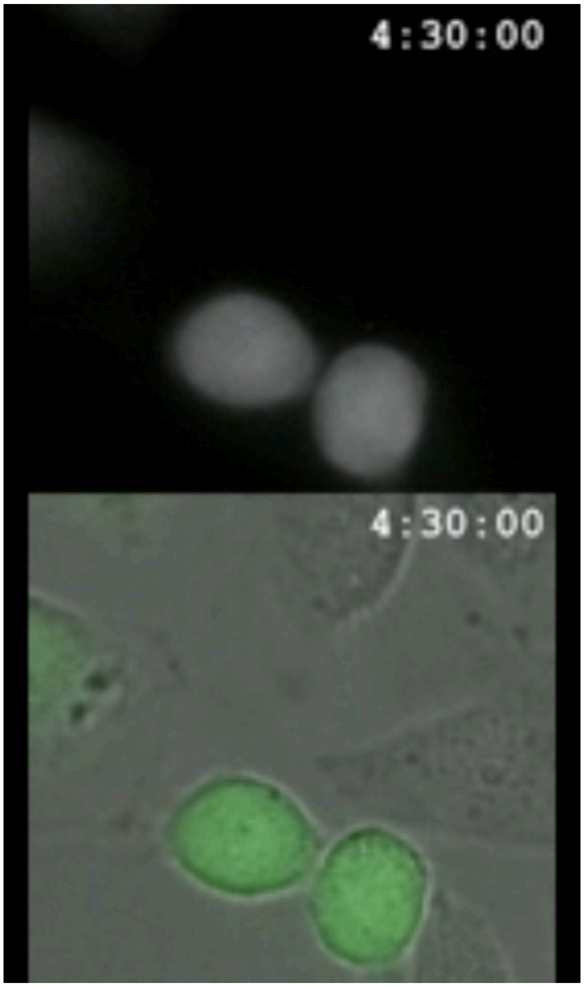

Supplementary Movie 3: Related to Figure 10B. MCF7 cells transfected with control shRNA were imaged using microscopy.

See Supplementary Movie 3

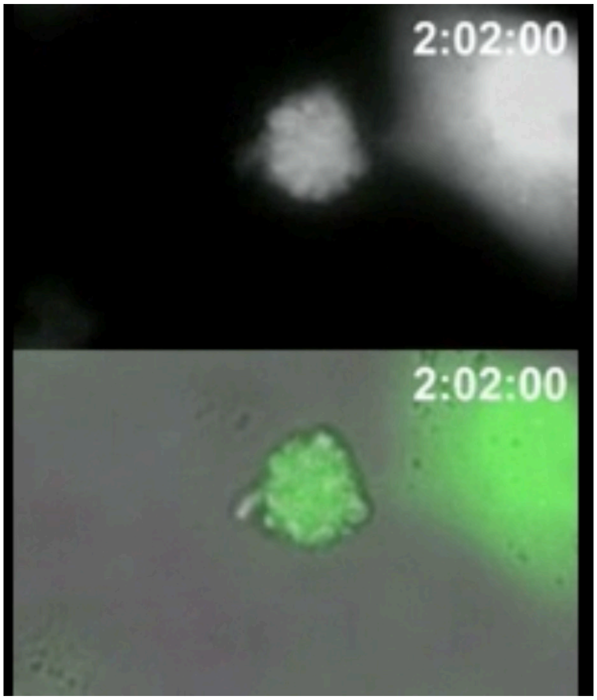

Supplementary Movie 4: Related to Figure 10B. MCF7 cells transfected with RRP15 shRNA were imaged using microscopy.

See Supplementary Movie 4
